# Exhaled Breath Temperature in COPD

**DOI:** 10.3390/medicina62101823

**Published:** 2026-09-22

**Authors:** Žarko Vrbica, Justinija Steiner, Davor Plavec

**Affiliations:** 1 Department for Health, University of Dubrovnik, 20000 Dubrovnik, Croatia; 2 Dubrovnik General Hospital, 20000 Dubrovnik, Croatia; 3 Osijek-Baranja County Health Center, 31000 Osijek, Croatia; 4 Prima Nova, Healthcare Institution, 10090 Zagreb, Croatia; 5 Medical Faculty, University JJ Stossmayer, 31000 Osijek, Croatia

**Keywords:** COPD, exhaled breath temperature, pre-COPD, smoking, aging

## Abstract

Chronic obstructive pulmonary disease (COPD) is a leading cause of morbidity and mortality worldwide. Identifying patients with early COPD remains challenging; however, even at this stage, substantial biological disease progression and irreversible lung injury may already be present. Considerable effort has therefore been directed toward identifying parameters that detect early pathophysiological changes before airflow limitation develops. The importance of this stage has recently been recognized and is described using terms such as “pre-COPD” and “early COPD”. Exhaled breath temperature (EBT) is a non-invasive method for detecting and monitoring respiratory inflammation. Most EBT studies have been conducted in asthma and have demonstrated the utility of this approach for assessing changes in airway inflammation. In patients with COPD, the number of airways and their vasculature is reduced, and EBT decreases proportionally with the degree of structural destruction; however, EBT may still increase during COPD exacerbations. In recent studies, the change in EBT after cigarette smoking in individuals without a COPD diagnosis significantly predicted disease progression after 2 years. Early interventions based on these findings should be tested for efficacy in preventing the development of overt COPD.

## 1. Chronic Diseases and Pre-Diseases

The natural course of many chronic progressive diseases is characterized by largely irreversible structural and functional changes once clinical criteria for diagnosis are fully met [1]. Consequently, conventional therapeutic interventions at later stages focus primarily on mitigating symptoms, slowing progression, and managing complications. Because active pathophysiological processes precede overt clinical disease, identifying these early changes offers a window for interventions—such as lifestyle or dietary modifications—that may prevent or delay disease onset. This paradigm underlies the established clinical frameworks for pre-hypertension and pre-diabetes [1]. Applying a similar preventative framework to smoking-related lung injury is essential, as detecting early, subclinical pathophysiological changes before the onset of irreversible airflow limitation remains a critical unmet need in respiratory medicine [2].

## 2. Chronic Obstructive Pulmonary Disease (COPD)

COPD is a major cause of morbidity and mortality worldwide. Available treatments can slow disease progression and reduce mortality, but they cannot reverse the pathophysiological processes that progressively destroy lung structure and function or restore already injured lung tissue [3]. Spirometry remains the diagnostic gold standard for COPD; however, it reflects integrated airflow limitation and lung-volume changes rather than directly measuring early small-airway or parenchymal injury. Because COPD-related lung damage begins in the small airways and subsequently extends to larger airways, substantial small-airway pathology may already be present by the time airflow limitation becomes detectable using conventional spirometry. Earlier intervention is therefore needed, and this requires a clinically useful definition of pre-COPD.

## 3. Pre-COPD

Pre-COPD has been proposed as a state identifying individuals with symptoms and/or functional or imaging abnormalities but preserved spirometry who are at increased risk of developing COPD. In Western countries, smoking is the most common cause of COPD. However, because not all smokers develop COPD, smoking exposure alone is insufficient to classify all smokers as having pre-COPD or to justify treatment intended to prevent smoking-related lung injury. Pre-COPD should therefore not be equated with smoking exposure alone. Rather, it refers to individuals with respiratory symptoms and/or functional or imaging abnormalities despite preserved spirometry, who are considered to be at increased risk of developing COPD. Several potential pre-COPD indicators have been associated with COPD development, although their associations with lung-function decline have been reported inconsistently. Smokers with symptoms had greater declines in lung function than smokers without symptoms, and a longer duration of chronic mucus hypersecretion was associated with greater concurrent forced expiratory volume in 1 s (FEV1) decline in some studies; however, these findings have not been consistent across studies. Preserved ratio impaired spirometry (PRISm) and low peak expiratory flow (PEF) were not associated with accelerated lung-function decline [4]. Chronic bronchitis symptoms are associated with excessive lung-function decline among smokers. Small-airway dysfunction is a precursor of COPD but is not detectable by standard spirometry until substantial destruction has occurred [5]. Computed tomography (CT)-assessed functional small-airway disease and emphysema have been associated with FEV1 decline among smokers, and individuals with spirometry-defined small-airways disease (SAD) have a faster decline in lung function than those without SAD; non-obstructive SAD is also more likely to progress to spirometry-defined COPD [6]. Smoking cessation and preventive counseling remain indicated for all smokers regardless of biomarker results. However, population screening using CT to detect SAD is not feasible. Detection of an acute inflammatory response to cigarette smoke may help identify smokers with a potentially higher-risk profile, but this should be distinguished from a validated diagnosis of pre-COPD or a definitive prediction of future COPD.

## 4. Inflammation in COPD

Important pathophysiological mechanisms involved in the development of COPD are well recognized. The most important is chronic inflammation caused by inhalation of harmful gases or particles. This chronic inflammation is characterized by an increased number of inflammatory cells, mainly located in pulmonary blood vessels, peripheral airways, and lung parenchyma. These cells release inflammatory mediators, including tumor necrosis factor alpha (TNF-α), interleukin (IL)-1, IL-6, chemokine ligand 8 (CXCL8), chemokine ligand 2 (CCL2), and matrix metalloproteinases (MMPs). This inflammation damages lung structure through two main pathological patterns: small-airway disease, due to peribronchiolar fibrosis, and emphysema, due to destruction of alveolar walls. These changes can be detected by CT, and most patients have mixed pathology that ultimately causes irreversible, progressive airflow limitation [7], as measured by spirometry. If airway inflammation could be detected and measured before spirometric changes occur, this could support identification of a pre-COPD state and enable appropriate preventive or therapeutic measures. Airway inflammation may precede spirometric airflow limitation and may contribute to future COPD development. However, detection of inflammation alone should be regarded as a potential risk marker rather than a validated definition of pre-COPD unless supported by longitudinal evidence of progression.

## 5. Measuring Lung Inflammation

Exhaled breath analysis includes several distinct approaches, including assessment of volatile organic compounds in exhaled air, markers measured in exhaled breath condensate, and physiological parameters such as exhaled breath temperature. Although these methods are promising, their clinical use in pre-COPD requires validation of sensitivity, specificity, reproducibility, and ability to predict future COPD. The main challenge is how to measure early inflammation in the lungs of patients at risk. The release of pro-inflammatory cytokines, such as IL-17A, IL-17F, IL-21, and IL-22, is associated with COPD progression and exacerbation of alveolar destruction; however, measurements in body fluids such as blood, plasma, or urine are not sufficiently sensitive or specific to represent the extent of lung inflammation. For this reason, increasing emphasis has been placed on measuring parameters in exhaled air, because many volatile substances in exhaled air are produced directly in the lungs and may provide information on the inflammatory lung profile. Patients with COPD have increased concentrations of H2O2, malondialdehyde, 4HNE (4-hydroxy-2-nonenal), 8-isoprostane, and ethane, a volatile product of lipid peroxidation, in exhaled breath condensate (EBC). These markers increase further during exacerbations; however, no exhaled-air inflammatory marker has yet been shown to be sufficiently sensitive and specific to detect a pre-COPD state and predict subsequent COPD development. EBC profiling shows clear promise for improving diagnosis, monitoring, and stratification in respiratory medicine, but its translation into clinical practice is hindered by limited standardization and validation [8,9]. Systemic inflammatory markers may not adequately reflect local airway inflammation in individual patients, whereas exhaled biomarkers may provide more direct information about airway processes. However, any candidate marker, including EBT, must demonstrate reproducibility, disease relevance, and longitudinal predictive value before it can be used to identify pre-COPD.

## 6. Lung Inflammation and Thermogenesis

Inflammation is a protective response elicited by tissue injury or destruction. In its acute form, it is characterized by the classical signs of pain (dolor), heat (calor), redness (rubor), swelling (tumor), and loss of function (functio laesa). Histologically, inflammation involves a complex sequence of events, including dilatation of arterioles, capillaries, and venules. Heat generation is an important feature of inflammatory processes. Indeed, the origin of the word inflammation (from the Latin inflammatio, inflammare, “to set on fire”) implies a heat-producing process [10]. Infections and/or immunologic mechanisms may trigger heat production in different disease states. The systemic response is referred to as fever and is associated with a complex sequence of events involving pyrogenic stimuli and neurogenic pathways that increase core body temperature. This has long been recognized as an important feature of different morbid conditions and has made temperature measurement and monitoring (thermometry) an integral part of standard physical examination. Systemic inflammation is associated with increased body temperature, which can be measured in the axilla, mouth, ear, or rectum, or using infrared devices. Local inflammatory events drive clinical symptoms in different organs. Some, such as joints in rheumatic diseases, are readily accessible to physical examination and increased local temperature can be directly assessed; others, such as the lungs and airways, are not accessible for direct assessment. Airway inflammation is considered a prominent feature of chronic respiratory diseases. If local temperature generated by inflammatory processes in the lungs could be measured, it might help detect inflammation caused by inhaled toxic substances at an early stage and support recognition of a pre-COPD pattern before lung damage causes spirometric changes. Because airway inflammation can alter bronchial blood flow and respiratory heat exchange, EBT is biologically plausible as an indirect marker of airway inflammatory activity. Nevertheless, this mechanistic rationale does not by itself establish EBT as a diagnostic or predictive tool for pre-COPD.

## 7. Measuring Exhaled Breath Temperature (EBT)

Airway inflammation in asthma is well studied, whereas inflammation in COPD and pre-COPD is less well characterized. Biomarkers such as fractional exhaled nitric oxide (FeNO), induced sputum eosinophils, and exhaled breath condensate have improved asthma phenotyping [11,12,13,14]. Because COPD is characterized by different inflammatory patterns, these biomarkers have limited applicability, and novel biomarkers of inflammation in COPD are needed [3].

The easiest way to approach inflammation in the bronchial tree is by analyzing the exhaled breath characteristics of the patient. Recognition that expired air contains clinically useful information dates to antiquity, when physicians including Hippocrates described characteristic odors, humidity, and warmth of a patient’s breath as indicators of systemic illness [15]. For centuries, however, breath analysis remained almost entirely qualitative. Physicians described the sweet odor of diabetic ketoacidosis, the fetor hepaticus associated with advanced liver disease, and the putrid breath characteristic of pulmonary abscesses, but temperature itself attracted little scientific attention. Unlike body temperature, expired air was considered too variable and too dependent upon environmental conditions to possess diagnostic value.

Modern breath analysis evolved during the twentieth century. Gas chromatography, mass spectrometry, infrared spectroscopy, and electrochemical sensing enabled increasingly sophisticated characterization of exhaled gases and volatile organic compounds [16]. By the 1980s and 1990s, interest had shifted toward identifying non-invasive biomarkers capable of replacing invasive procedures such as bronchoscopy or induced sputum analysis. A major milestone occurred with the introduction of fractional exhaled nitric oxide (FeNO) as a marker of eosinophilic airway inflammation [11,12]. The rapid clinical acceptance of FeNO demonstrated that exhaled breath could provide reproducible physiological information relevant to disease management. This success created an intellectual environment in which other breath-derived biomarkers, including exhaled breath condensate, volatile organic compounds, and eventually exhaled breath temperature, could be seriously investigated [12,17].

The concept of measuring breath temperature emerged much later. Measuring the exhaled breath temperature offers an attractive possibility because it requires only tidal breathing, is completely non-invasive, inexpensive, and potentially suitable for repeated bedside or home monitoring. Unlike biochemical biomarkers, EBT measures a physiological consequence of airway inflammation (enhanced bronchial vascular perfusion) rather than inflammatory mediators themselves.

A landmark study by Paredi and colleagues demonstrated that patients with asthma exhibited a faster rise in exhaled breath temperature than healthy controls [18]. More importantly, inhaled corticosteroid therapy reduced this abnormal thermal profile, suggesting that EBT reflected a reversible component of airway inflammation rather than fixed anatomical characteristics. This elegant observation transformed EBT from a physiological curiosity into a potential biomarker of inflammatory airway disease.

This suggested that airway inflammation increases bronchial blood flow, thereby warming expired air [19]. Since then, multiple clinical studies have explored EBT in asthma, COPD, cystic fibrosis, bronchiectasis, smoking-related lung disease, and environmental exposure. These discoveries demonstrated that exhaled air represented not merely waste gas but a complex physiological product reflecting metabolic and pulmonary function [18].

With every breath, inspired air is warmed from ambient temperature to approximately 37 °C while simultaneously becoming fully saturated with water vapor before reaching the distal airways. The warming and humidification of inspired air occur predominantly within the nose and upper airways under resting conditions. During exercise, mouth breathing, or exposure to cold environments, however, progressively larger portions of this task are transferred to the trachea and bronchial tree. Consequently, the bronchial circulation assumes a critical role in maintaining thermal homeostasis throughout the respiratory tract. It is now recognized that the bronchial circulation actively participates in airway defense, inflammatory responses, tissue repair, and thermoregulation. During expiration, a substantial proportion of this heat is transferred back from the airway mucosa to the outgoing gas, generating a measurable exhaled breath temperature that reflects the complex interaction between airway structure, bronchial circulation, pulmonary perfusion, ventilation patterns, and environmental conditions. These data are confirmed by direct mucosal and air temperature measurements throughout the bronchial tree in a delicate study by Khosravi M. et al. [20]. Inflammation increases bronchial vascular permeability and angiogenesis, both of which enhance heat transfer from airway tissues to exhaled gas. Unlike the pulmonary circulation, whose principal function is gas exchange, the bronchial circulation supplies oxygenated blood to the conducting airways and plays a critical role in warming and humidifying inspired air. Experimental studies have demonstrated that bronchial blood flow increases markedly during airway inflammation and contributes substantially to airway edema, vascular permeability, and tissue remodeling. Histological studies in asthma further revealed pronounced angiogenesis and enlargement of bronchial vessels, suggesting that alterations in airway vascularity might influence airway thermodynamics.

Bronchial blood flow is highly dynamic. Experimental studies have demonstrated increases of more than two-fold following inflammatory stimulation, exercise, inhalation of cold air, allergen challenge, and administration of vasoactive mediators. Such rapid vascular adaptation allows the respiratory tract to maintain thermal stability under widely varying environmental conditions. These physiological observations prompted investigators to hypothesize that inflammatory airway diseases might be associated with measurable alterations in exhaled breath temperature. The hypothesis was appealing because it linked an easily measurable physical parameter with one of the fundamental pathological processes underlying chronic airway inflammation.

Unlike biochemical biomarkers, which quantify inflammatory mediators directly, EBT measures one of the physiological consequences of inflammation. Airway inflammation induces vasodilation, angiogenesis, and increased bronchial blood flow, thereby enhancing heat transfer from bronchial tissues to inspired and expired air.

During the past two decades, investigators have explored EBT in a broad spectrum of respiratory disorders. Most research has focused on asthma, where elevated EBT has consistently been associated with disease activity, airway vascularity, and response to anti-inflammatory therapy. Additional studies have examined COPD [21], bronchiectasis, cystic fibrosis, acute respiratory infections, environmental pollution, tobacco exposure, and occupational lung diseases. More recently, advances in sensor technology have stimulated interest in portable devices capable of home monitoring, while the emergence of digital health platforms has prompted renewed consideration of EBT as a component of integrated respiratory monitoring [22].

One of the earliest pathological events during airway inflammation is vascular activation, and EBT may serve as an early marker of disease activity before symptoms or lung-function decline occur. However, EBT has not yet entered mainstream clinical use because of several important practical limitations. First, measurement techniques vary considerably among studies, environmental conditions substantially influence recorded values, and standardized operating procedures remain incompletely established. Furthermore, unlike FeNO, which reflects a relatively specific inflammatory pathway involving inducible nitric oxide synthase and type 2 inflammation, EBT represents the net effect of multiple physiological processes, both inflammatory and non-inflammatory. Consequently, interpretation requires careful consideration of numerous biological and technical variables.

There is also the problem with the standardization of the way how the EBT should be measured. The simplest measurement is the plateau temperature, defined as the maximum stable temperature reached during prolonged expiration or repeated tidal breathing. This value approximates the equilibrium temperature of exhaled air under standardized conditions. Plateau temperature is relatively simple to measure and generally exhibits good short-term reproducibility. However, because it represents an equilibrium value, it may be less sensitive to subtle physiological changes occurring during the early phase of expiration.

The original methodology emphasized the rate of temperature increase during expiration rather than the absolute plateau temperature. The pioneering studies by Paredi and colleagues focused on the rate of temperature increase during expiration [18]. The physiological rationale was elegant. If airway inflammation increases bronchial blood flow, then exhaled air should warm more rapidly as it passes through the conducting airways. This parameter therefore reflects the kinetics of airway heat exchange rather than its final equilibrium. Several studies have suggested that the rate of temperature increase may be more sensitive than plateau temperature for detecting airway inflammation. This distinction reflected the belief that the kinetics of airway warming were more sensitive to changes in bronchial perfusion than the final equilibrium temperature. Subsequent studies, however, explored both parameters and demonstrated that each might provide complementary physiological information.

An important contribution came from Popov and coworkers, who systematically investigated the determinants of EBT and emphasized the importance of standardized measurement conditions. Their work demonstrated that environmental temperature, breathing pattern, smoking status, and technical aspects of measurement substantially influenced recorded values. These studies highlighted one of the central challenges facing EBT research: the need to distinguish biological variability from methodological variability. Future research should define which EBT endpoint is relevant to each aspect of pathophysiology: absolute plateau temperature, rate of temperature rise, or within-individual change after a stimulus such as cigarette smoking. These endpoints may reflect different physiological processes and may not be interchangeable. Their interpretation is further limited by the absence of standardized measurement protocols and by the non-specific nature of EBT as a marker of respiratory heat exchange.

Despite these efforts, no internationally accepted guideline comparable to the ATS/ERS recommendations for spirometry or FeNO currently exists. This lack of consensus represents one of the major barriers to regulatory approval and widespread clinical implementation. Several barriers prevent widespread clinical implementation. The most important is the absence of universally accepted measurement protocols with limited device standardization and substantial overlap between healthy and diseased populations.

Advances in measurement technology with faster-response thermistors, improved digital signal processing, automatic identification of temperature plateaus, and standardized breathing protocols make EBT more precise in inflammation measurement. Portable handheld instruments became available, allowing measurements to be performed in outpatient clinics and, eventually, in patients’ homes. These developments significantly improved practicality and reproducibility.

Nevertheless, technological evolution also introduced an important challenge. Different investigators employed different devices, sampling frequencies, breathing maneuvers, averaging algorithms, and definitions of the measured parameter. Some studies reported maximum plateau temperature, whereas others analyzed the slope of temperature increase or calculated composite indices derived from repeated respiratory cycles. This lack of methodological uniformity complicates direct comparison among published studies and remains one of the principal obstacles to clinical implementation.

The modern approach has shifted from static assessment to dynamic monitoring of EBT, following changes in the patient’s airway thermal profile as a surrogate of underlying inflammation. This shift from a static biomarker to a dynamic physiological signal could represent the next stage in the evolution of exhaled breath temperature. Repeated measurements might provide information unavailable from isolated clinic assessments. Before symptoms develop, detection of subtle evidence of increasing airway inflammation and vascular activation may improve treatment strategies.

During the first decade of EBT research, asthma remained the principal focus. Asthma remains the disease in which EBT has been investigated most extensively. Numerous studies demonstrate elevated EBT compared with healthy subjects, correlation of EBT with airway inflammation and reduction in EBT following inhaled corticosteroid treatment. Acute asthma exacerbations provide a unique opportunity to evaluate EBT as a dynamic biomarker because during exacerbations airway inflammation increases rapidly with the increased bronchial blood flow and vascular permeability. Several studies have reported increased EBT during exacerbations, followed by normalization after treatment. These findings suggest that EBT may reflect short-term inflammatory activity and that EBT is fundamentally a physiological biomarker of airway vascular function.

Piacentini and colleagues extended EBT research into pediatric asthma [23,24]. Children represented an attractive population because conventional inflammatory assessments such as induced sputum are often difficult to perform, whereas EBT requires only quiet tidal breathing. Their studies demonstrated that EBT measurements were feasible even in young children and suggested that elevated values correlated with asthma activity. These findings reinforced the potential role of EBT as a completely non-invasive biomarker suitable for repeated monitoring throughout childhood.

Interest subsequently expanded beyond asthma. COPD is characterized by persistent airway inflammation involving neutrophils, macrophages, CD8+ lymphocytes, and structural airway cells. Chronic exposure to cigarette smoke induces endothelial dysfunction, oxidative stress, microvascular injury, and remodeling of the bronchial circulation. Simultaneously, repeated episodes of acute inflammation promote transient increases in bronchial blood flow like those observed in asthma.

Most published studies demonstrate modestly elevated EBT in patients with stable COPD compared with healthy non-smokers [22]. However, the magnitude of these differences is generally smaller than that reported in asthma. Studies have demonstrated increased EBT during acute exacerbations, although the results are less consistent than in asthma. Several investigators have reported transient increases in EBT during exacerbations followed by partial normalization after successful treatment. This observation parallels the findings in asthma and supports the concept that airway inflammation produces measurable changes in respiratory heat exchange regardless of the underlying disease. Smoking influences EBT through complex mechanisms involving airway inflammation, vascular dysfunction, and chronic structural remodeling. Current and former smokers often demonstrate altered EBT values, although findings vary between studies [22]. The variability of the study results probably reflects the complex pathophysiology of COPD, in which chronic inflammation coexists with emphysematous destruction, altered airway geometry, smoking-related vascular dysfunction, and impaired pulmonary perfusion. Nevertheless, several investigations suggest that EBT increases during periods of heightened inflammatory activity, supporting the concept that airway thermodynamics respond to multiple forms of respiratory inflammation.

Researchers also explored EBT in bronchiectasis, cystic fibrosis, allergic rhinitis, acute respiratory infections, occupational exposure to airborne pollutants, and cigarette smoking. Although the magnitude of observed changes varied among studies, a common pattern emerged: diseases characterized by active airway inflammation generally exhibited higher EBT than healthy individuals.

Measurement of exhaled breath temperature (EBT) during multiple tidal breaths provides a simple, non-invasive tool for monitoring airway inflammation [25]. Devices that measure exhaled breath temperature have been available for some time. The most commonly used approach is a multiple-breath procedure using the portable X-halo device (Delmedica Investments Pvt Ltd., Singapore), in which expired thermal energy is accumulated in an insulated vessel containing a heat sink with high thermal capacity, making measurements less dependent on ambient temperature. Evidence suggests that, when an appropriate procedure is used, lung-related thermal information can be measured independently of body temperature or external conditions. No association was found between EBT and ambient conditions, including room temperature, atmospheric pressure, and humidity. Although otic and axillary temperatures were highly correlated with each other, EBT did not show a meaningful association with either [10]. Because EBT values lie within a narrow range, thermometers designed for this purpose must be precise and highly sensitive [26]. Evidence indicates that changes associated with the activity of different lung diseases, mainly asthma but also malignancies and other pathophysiological processes, can be assessed [27]. EBT has been well studied in patients with asthma, in whom values were higher than in controls. Anti-inflammatory treatment reduced EBT in patients with asthma, correlating well with improvements in spirometry [10]. A limitation of EBT is that these changes are small, and no standard reference values have been established because of the substantial overlap between groups. In patients with asthma, evidence suggests that within-individual increases in EBT may predict the onset of exacerbation before symptoms or spirometric changes occur.

## 8. EBT in COPD

In COPD, evidence indicates that fluctuations in exhaled breath temperature are associated with disease extent and exacerbations. A statistically significant difference has been reported between smokers and non-smokers [28]. In COPD, within-individual increases in EBT are associated with disease worsening and can be detected during exacerbations, with good correlation with sputum leukocyte count. Although EBT differs significantly between healthy smokers and non-smokers, the temperature ranges overlap, preventing establishment of a clear reference range for normal and pathological findings.

## 9. Inflammation in Smokers with Pre-COPD

Although smoking is a major environmental risk factor for COPD, only a proportion of smokers develop the disease [29]. Because some smokers develop COPD whereas others do not, it has been hypothesized that the inflammatory response to inhaled smoke differs among individuals. Barnes et al. [28] showed that patients with COPD had a more severe inflammatory response than smokers without airway obstruction. This may reflect genetic and epigenetic differences in individual responses to smoking. It may be hypothesized that smokers who subsequently develop COPD mount an inflammatory response to each cigarette smoked, whereas those who do not develop COPD have a less pronounced inflammatory reaction. If so, measurement of EBT after cigarette smoking may detect inflammation-related changes and help identify smokers at increased risk of developing COPD in the future.

## 10. EBT in Smokers

It is already known that EBT increases after cigarette smoking and progressively decreases as the time since the last cigarette increases. Thus, EBT appears to be increased in smokers and sensitive to the acute effects of cigarette smoke [26,27]. Recent findings have shown that some smokers respond with an increase in EBT after smoking a cigarette, whereas others do not [30].

## 11. EBT and Pre-COPD

The change in EBT after smoking a cigarette in individuals without a diagnosis of COPD was significantly predictive of disease progression after 2 years [27]. The change in EBT after smoking a cigarette at the initial visit was significantly predictive of disease progression, defined as newly diagnosed COPD or newly diagnosed COPD plus severity progression, after 2 years. An increase in EBT after acute exposure to cigarette smoke could represent a marker of pathophysiological changes consistent with pre-COPD before lung function declines. EBT may detect the inflammatory reaction to cigarette smoke that ultimately contributes to overt COPD. This hypothesis should be prospectively tested in larger studies; if confirmed, early interventions based on these findings should be evaluated for efficacy in preventing overt COPD. Table 1 summarizes existing evidence on EBT in COPD/pre-COPD, including populations, sample sizes, measurement methodology, outcomes, effect direction, and major limitations.

## 12. Conclusions

Identifying pathophysiological changes that lead to COPD development in at-risk individuals may enable earlier intervention, appropriate preventive or therapeutic measures, and prevention of irreversible changes and destruction of lung tissue and airways. The inflammatory response to noxious inhaled particles is an important mechanism of COPD progression. Measuring this inflammation may help identify susceptible individuals. To achieve this, a simple, affordable, and easy-to-use method or device is needed to detect airway inflammation. Measuring the rise in EBT after cigarette smoking in smokers without COPD may be one approach to identifying smokers who are more likely to develop COPD if they continue to smoke. Because current evidence remains limited, the scientific community should further explore the potential role of EBT in pre-COPD, and additional studies are needed.

## Figures and Tables

**Table 1:** EBT studies in COPD/pre-COPD, including populations, sample sizes, measurement methodology, outcomes, effect direction and major limitations.

Study/Status	Population	N	Measurement Methodology	Key Outcomes	Effect Direction *	Major Limitations
COPD|Lázár et al., 2014|peer-reviewed [21]	Stable COPD; AECOPD measured at onset and after recovery; smoking/ex-smoking and never-smoking controls	75 total: 20 stable COPD; 17 AECOPD; 19 smoking/ex-smoking controls; 19 never-smoking controls	X-halo; peak EBT during multiple tidal breaths; repeatability in non-smokers; sputum in AECOPD	Stable COPD lower than smoking/ex-smoking controls. AECOPD higher than stable condition; EBT fell after recovery. At onset, EBT correlated with sputum leukocyte count and neutrophil percentage.	Stable COPD ↓; AECOPD ↑; recovery ↓	Small groups; age differed across groups; single-centre; peak EBT only; limited longitudinal follow-up beyond exacerbation recovery.
COPD|García et al., 2012|ERS abstract [31]	COPD across GOLD 2011 A–D groups and healthy controls	160: 80 COPD; 80 controls	X-halo; EBT with spirometry, CAT and exacerbation history	No overall COPD-control difference. Higher EBT in patients with previous exacerbations and in GOLD C/D than B. No meaningful association with FEV1% or CAT reported.	Overall ↔; exacerbation-prone ↑	Conference abstract; unclear control matching and environmental protocol; cross-sectional; prior exacerbation history may confound phenotype.
COPD|Tufvesson et al., 2020|peer-reviewed [25]	Asthma, COPD, systemic sclerosis and healthy controls	65 total: 18 COPD; 16 asthma; 17 SSc; 14 healthy	Novel fractional EBT: Tmax, central T1max, peripheral T3max and T3/T1; FeNO, spirometry, oscillometry, plethysmography, DLCO	T3/T1 lower in COPD or SSc than asthma. EBT variables related to lung volumes; peripheral temperature related to DLCO % predicted. Large overlap between groups.	Peripheral/central ratio ↓ in COPD vs. asthma	Small diagnostic groups; exploratory multiple comparisons; large overlap; COPD vs. healthy discrimination not established; novel device limits comparability.
COPD|Gawlik et al., 2014|abstract [32]	Stable asthma and COPD; healthy controls; recent infection/exacerbation excluded	154 total: 124 asthma/COPD combined; 30 controls	X-halo; mean of 3 measurements performed one day apart; controlled room temperature/humidity	EBT lower in chronic obstructive respiratory disease and asthma than controls; inverse correlation between age and EBT.	Disease groups ↓; age association ↓	Abstract only; asthma and COPD subgroup sizes/results not separated; age confounding likely; unusual repeated-day protocol.
PRE-COPD|Labor et al., 2015 MARKO|ERS preliminary abstract [33]	Smokers > 20 pack-years, age 40–65, no prior COPD diagnosis; reassessed after 2 years	122 reassessed	EBT before and after a smoked cigarette; lung function, CAT and SGRQ; COPD reassessment	ΔEBT associated with quality of life and marginally with progression to COPD.	Predictive association reported; exact direction not safely extractable from abstract	Preliminary subset; conference abstract; cigarette challenge is difficult to standardise and unsuitable for ex-smokers/non-smokers; no validated threshold.
PRE-COPD|Vrbica et al., 2023 MARKO|peer-reviewed longitudinal [30]	Smokers/ex-smokers > 20 pack-years, age 40–65, at risk for COPD	320 reassessed after mean 2.1 years	Baseline and follow-up spirometry and EBT; in active smokers post-cigarette ΔEBT; questionnaires, hsCRP and 6MWT	Questionnaire domains plus male sex predicted incident COPD (AUC 0.79). In active smokers, adding ΔEBT increased model AUC to 0.83.	Incremental model performance ↑	EBT contribution evaluated within a multivariable model, not as stand-alone test; internal cohort analysis; cigarette challenge; no external validation or clinical cut-off.

* Effect arrows refer to EBT unless otherwise stated. Arrow UP means that the EBT is increased. Arrow DOWN means that the EBT is decreased.

## Data Availability

No new data were created or analyzed in this study. Data sharing is not applicable to this article.

## Abbreviations

The following abbreviations are used in this manuscript:

COPD	Chronic obstructive pulmonary disease
PRISm	Preserved ratio impaired spirometry
FEV1	Forced expiratory volume in 1 s
PEF	Peak expiratory flow
CT	Computer tomography
SAD	Small airways disease
EBC	Exhaled breath condensate
EBT	Exhaled breath temperature

## References

[1] Khosravi A., Gharipour M., Nezafati P., Khosravi Z., Sadeghi M., Khaledifar A., Taheri M., Golshahi J., Sarrafzadegan N. (2017). Pre-hypertension, pre-diabetes or both: Which is best at predicting cardiovascular events in the long term?. J. Hum. Hypertens..

[2] Plavec D., Vrbica Ž. (2024). What is pre-COPD and do we know how to treat it?. Expert Rev. Respir. Med..

[3] Global Initiative for Chronic Obstructive Lung Disease (GOLD) (2025). Global Strategy for the Diagnosis, Management, and Prevention of Chronic Obstructive Pulmonary Disease (2025 Report). http://www.goldcopd.org.

[4] Fan J., Cong S., Zhang Y., Jiang X., Wang N., Fang L., Chen Y. (2025). Associations of pre-COPD indicators with lung function decline and their longitudinal transitions. Pulmonology.

[5] Spittle D.A., Thomas M., Stevens C., Gazwani A., Fenton S., De Soyza J., Turner A.M. (2024). Symptoms of COPD in the absence of airflow obstruction are more indicative of pre-COPD than overdiagnosis. ERJ Open Res..

[6] Zhou Y., Wu F., Deng Z., Wang Z., Tian H., Huang P., Zheng Y., Yang H., Zhao N., Dai C. (2025). Lung function decline and incidence of chronic obstructive pulmonary disease in participants with spirometry-defined small airway dysfunction: A 15-year prospective cohort study in China. Respir. Res..

[7] Xu J., Zeng Q., Li S., Su Q., Fan H. (2024). Inflammation mechanism and research progress of COPD. Front. Immunol..

[8] Malerba M., Purghè B., Ragnoli B., Manfredi M., Baldanzi G. (2025). Molecular profiling of exhaled breath condensate in respiratory diseases. Ann. Med..

[9] Freund R., Sauvain J.J., Suarez G., Wild P., Charreau T., Debatisse A., Sakthithasan K., Jouannique V., Pralong J.A., Guseva Canu I. (2024). Discriminative potential of exhaled breath condensate biomarkers with respect to chronic obstructive pulmonary disease. J. Occup. Med. Toxicol..

[10] Popov T.A., Dunev S., Kralimarkova T.Z., Kraeva S., DuBuske L.M. (2007). Evaluation of a simple, potentially individual device for exhaled breath temperature measurement. Respir. Med..

[11] Dweik R.A., Boggs P.B., Erzurum S.C., Irvin C.G., Leigh M.W., Lundberg J.O., Olin A.-C., Plummer A.L., Taylor D.R. (2011). on behalf of the American Thoracic Society Committee on Interpretation of Exhaled Nitric Oxide Levels (FeNO) for Clinical Applications. An Official ATS Clinical Practice Guideline: Interpretation of Exhaled Nitric Oxide Levels (FeNO) for Clinical Applications. Am. J. Respir. Crit. Care Med..

[12] Horváth I., Barnes P.J., Loukides S., Sterk P.J., Högman M., Olin A.-C., Amann A., Antus B., Baraldi E., Bikov A. (2017). A European Respiratory Society technical standard: Exhaled biomarkers in lung disease. Eur. Respir. J..

[13] Kharitonov S.A., Barnes P.J. (2006). Exhaled biomarkers. Chest.

[14] Horváth I., Hunt J., Barnes P.J. (2005). Exhaled breath condensate: Methodological recommendations and unresolved questions. Eur. Respir. J..

[15] Amann A., Smith D. (2013). Volatile Biomarkers: Non-Invasive Diagnosis in Physiology and Medicine.

[16] Miekisch W., Schubert J.K., Noeldge-Schomburg G.F. (2004). Diagnostic potential of breath analysis—focus on volatile organic compounds. Clin. Chim. Acta.

[17] Popov T.A., Kralimarkova T.Z., Dimitrov V.D. (2012). Measurement of exhaled breath temperature in science and clinical practice. Breathe.

[18] Paredi P., Kharitonov S.A., Barnes P.J. (2002). Faster rise of exhaled breath temperature in asthma: A novel marker of airway inflammation?. Am. J. Respir. Crit. Care Med..

[19] Paredi P., Kharitonov S.A., Barnes P.J. (2005). Correlation of exhaled breath temperature with bronchial blood flow in asthma. Respir. Res..

[20] Khosravi M., Lin R.-L., Maskey A.P., Pandey S., Lin A.-H., Lee L.-Y. (2021). A Distinct Difference Between Air and Mucosal Temperatures in Human Respiratory Tract. Front. Med..

[21] Lázár Z., Bikov A., Martinovszky F., Gálffy G., Losonczy G., Horváth I. (2014). Exhaled breath temperature in patients with stable and exacerbated COPD. J. Breath Res..

[22] Popov T.A., Kralimarkova T.Z., Labor M., Plavec D. (2017). The added value of exhaled breath temperature in respiratory medicine. J. Breath Res..

[23] Piacentini G.L., Peroni D., Crestani E., Zardini F., Bodini A., Costella S., Boner A.L. (2007). Exhaled air temperature in asthma: Methods and relationship with markers of disease. Clin. Exp. Allergy.

[24] Ferrante G., Tenero L., Zaffanello M., Piazza M., Piacentini G. (2026). The evolution of scientific knowledge in childhood asthma over time. Eur. Respir. Rev..

[25] Tufvesson E., Nilsson E., Popov T.A., Hesselstrand R., Bjermer L. (2020). Fractional exhaled breath temperature in patients with asthma, chronic obstructive pulmonary disease, or systemic sclerosis compared to healthy controls. Eur. Clin. Respir. J..

[26] Carpagnano G.E., Ruggieri C., Scioscia G., Storto M.M., Zoppo L., Foschino-Barbaro M.P. (2016). Is the Exhaled Breath Temperature Sensitive to Cigarette Smoking?. COPD.

[27] Labor M., Vrbica Ž., Gudelj I., Labor S., Jurić I., Plavec D. (2016). Exhaled Breath Temperature as a Novel Marker of Future Development of COPD: Results of a Follow-Up Study in Smokers. COPD.

[28] Barnes P.J. (2016). Inflammatory mechanisms in patients with chronic obstructive pulmonary disease. J. Allergy Clin. Immunol..

[29] Yang I.A., Jenkins C.R., Salvi S.S. (2022). Chronic obstructive pulmonary disease in never-smokers: Risk factors, pathogenesis, and implications for prevention and treatment. Lancet Respir. Med..

[30] Vrbica Ž., Steiner J., Labor M., Gudelj I., Plavec D. (2023). Breathlessness and “exacerbation” questions predictive for incident COPD (MARKO study): Data after two years of follow-up. PeerJ.

[31] García G., Bergna M., Jové O.L. (2012). Exhaled breath temperature in COPD patients. Eur. Respir. J..

[32] Gawlik R., Rajzer-Wrobel M., Ziora D., DuBuske L.M. (2014). Correlation of exhaled breath temperature with age in chronic respiratory diseases. J. Allergy Clin. Immunol..

[33] Labor M., Plavec D. (2015). Exhaled breath temperature as a possible early marker in smokers at risk for COPD (MARKO study). Eur. Respir. J..

